# The Effects of Tea Polyphenols on the Emulsifying and Gelling Properties of Minced Lamb After Repeated Freeze–Thaw Cycles

**DOI:** 10.3390/foods14132259

**Published:** 2025-06-26

**Authors:** Xueyan Yun, Ganqi Yang, Limin Li, Ying Wu, Xujin Yang, Aiwu Gao

**Affiliations:** 1 College of Food Science and Engineering, Inner Mongolia Agricultural University, Hohhot 010018, China; yun_imau@163.com (X.Y.); 13474927598@163.com (G.Y.); 13848441713@163.com (L.L.); 2 Department of Agricultural and Environmental Sciences, Tennessee State University, State of Tennessee, Nashville, TN 37290, USA; ywu@tnstate.edu

**Keywords:** tea polyphenols, freeze–thawed minced lamb, emulsifying properties, gelling properties, protein conformation

## Abstract

Minced lamb remains one of the most produced meat products in the meat industry, across both the food service and retail sectors. Tea polyphenols (TPs), renowned for their diverse biological activities, are increasingly being employed as natural food additives in research and development. Tea polyphenols at concentrations of 0.00% (CG), 0.01% (TP1), 0.10% (TP2), and 0.30% (TP3) were added to lamb which had undergone a series of freeze–thaw cycles. The presence of tea polyphenols led to a significant decrease in the number of disulfide bonds, resulting in a slower oxidation rate. In addition, the surface hydrophobicity and juice loss of the minced lamb supplemented with tea polyphenols were 91.23 ± 0.22 and 20.00 ± 0.46, respectively, representing a reduction of 1.5% and 7.59% compared to the group without the addition of tea polyphenols. However, the addition of high-dose tea polyphenols also led to a reduction in emulsification stability, alterations in protein conformation, and changes in water migration. Furthermore, the incorporation of a minimal quantity of tea polyphenols (0.01%) resulted in enhanced emulsification stability, water retention, textural properties, and microstructures in minced lamb. This suggests that tea polyphenols have the potential to improve the quality of minced lamb following freezing and thawing processes.

## 1. Introduction

Minced meat is an oft-consumed food product due to its convenience in purchasing and handling and its richness in flavor and nutrients [1]. Presently, minced meat products are primarily transported and stored while frozen [2]. During the products’ processing, storage, transportation, and consumption, the minced meat may undergo a few freezing and thawing cycles [3], resulting in the oxidation of fats and proteins in the minced meat [4]. Furthermore, ice crystals formed during the freezing process may result in the rupture of the cellular structure [5], thereby altering the nature of the minced meat gel and, consequently, affecting the commercial value of the minced meat [6].

A substantial body of research has been dedicated to the development of efficacious antifreeze agents that can ensure optimal food quality during frozen storage, minimize the potential damage to biological tissues from ice crystals, effectively resist fat oxidation, and delay the process of protein denaturation [5]. It was discovered that the addition of 280 mg/kg sodium pyrophosphate and 440 mg/kg sodium tripolyphosphate could impede the denaturation of grass carp muscle fiber proteins at low temperatures [7]. Sucrose and sorbitol, widely used as commercial cryoprotectants, inhibit ice crystal formation via their water-binding hydroxyl groups. While cost-effective, their cryoprotective efficacy against freeze-induced crystallization, recrystallization, and structural damage remains limited. Additionally, elevated doses may increase the heat and sugar content of products, adversely affecting their quality [8]. Moreover, the utilization of enhanced doses may result in products with augmented heat and a higher sugar content, which has a detrimental impact on the product’s quality [9].

In order to address the limitations of existing antifreezing agents, the current research aims to improve the freeze–thaw phenomenon in minced meat by using natural substances [10], with a particular focus on polyphenols. For example, the addition of a lyophilized soy lecithin liposome mixture containing 6.4 g alginate effectively improved the water retention of cod myosin gels [11]. The addition of 0.7% (*w*/*w*) young apple polyphenols slowed myofibrillar protein oxidation and protected the structure of myofibrillar protein [12]. The addition of 0.75% Masala extract resulted in a notable reduction in lipid oxidation and microbial growth in minced tilapia sausages [13]. Similarly, the maceration of herring mince with a mixture of 2% rosemary extract containing ascorbic acid, α-tocopherol, and citric acid had an effect of extending the oxidative lag period [14].

Tea polyphenols (TPs), extracted from tea leaves, are being increasingly applied in meat and meat products as replacements for potentially hazardous artificial antioxidants. This shift is driven by their trifecta of advantages: low cost, safety, and high efficiency [10]. Tea polyphenols are a complex of polyhydroxyphenolic compounds in tea, consisting of more than 30 phenolic substances, which exhibit antioxidant, antibacterial, mold-inhibiting, and other physiological activities [15]. These properties have led to the use of tea polyphenols as an antifreeze, preservative, and antioxidant in the processing of frozen fish and meat products. For example, tea polyphenols can effectively attenuate the lipid oxidation for Antarctic white shrimps during freezing [16], as well as in cold-stored scallops [17]. In addition, it has also been reported that tea polyphenols can prevent the oxidation and denaturation of chub myosin over a wide range of fluctuating frozen-storage conditions [18]. Additionally, tea polyphenols have also demonstrated effects such as inhibiting protein denaturation and improving the thermal stability of muscle proteins in carp meat [19].

Previous studies on tea polyphenols in frozen–thawed products mainly focused on quality changes after limited cycles, while neglecting their effects on the emulsion stability and gel properties of lamb mince under repetitive freezing–thawing. This study prepared a polyphenol–meat composite by adding different concentrations of tea polyphenols to minced lamb, aiming to examine how freeze–thaw cycles impact protein conformation, emulsion stability, and gel properties.

## 2. Materials and Methods

### 2.1. Materials and Reagents

In this experiment, 3- to 6-month-old Sunit lamb leg meat was utilized, sourced from the Sunit Lamb Specialty Store (Hohhot, China). The tea polyphenols (99% purity) were supplied by Shanghai Maclean Biochemical Co., Ltd. (Shanghai, China). Tris (hydroxymethyl) aminomethane (TRIS) was provided by Beijing Solarbio Science & Technology Co., Ltd. (Beijing, China). All of the chemicals (analytical reagent) were purchased from Sinopharm Chemical Reagent Co. (Tianjin, China).

### 2.2. Preparation of Minced Meat

**Figure 1:**
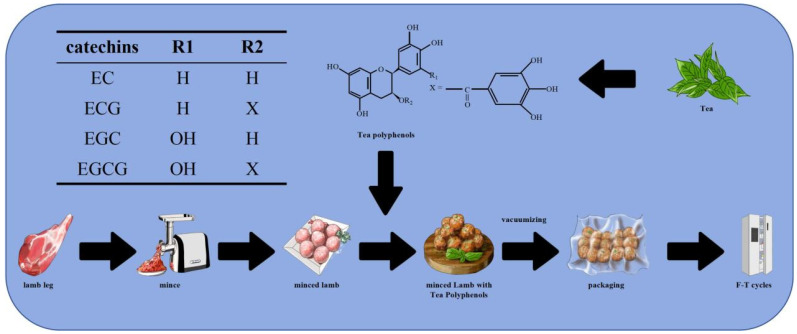
Structure of tea polyphenols (TPs) and preparation of minced lamb with tea polyphenols.

The minced lamb was prepared using a meat grinder (JYS-A960, Joyoung Co., Ltd., Shandong, China) equipped with a 3 mm diameter grinder plate, and the grinding process was conducted for 2 min at room temperature (25 ± 1 °C). After grinding, the following ingredients were added to the meat while mixing sodium chloride (2%) and ice water (10%), and fat (removed from the lamb) was also added, as shown in Figure 1. Tea polyphenols were added at various concentrations for different treatment groups, namely 0.00% (control group (CG)), 0.01% (*w*/*w*) for one tea polyphenol group (TP1), 0.10% (*w*/*w*) for another (TP2), and 0.30% (*w*/*w*) (TP3), respectively.

### 2.3. Freeze–Thaw Treatment

The minced lamb was divided into 24 portions at 5 g per portion. All portions were vacuum-packed using a vacuum packaging machine (DZ-300N, Shanghai Qingpa Packaging Machinery Co., Ltd., Shanghai, China). The samples were first cooled in a refrigerator (BCD-321WDBA, Qingdao Haier Co., Ltd., Qingdao, China) at 4 °C for 24 h, then frozen at −18 °C for 24 h, and then thawed at 4 °C for 24 h. This process was repeated 1, 3, 5, 7 and 9 times, respectively.

### 2.4. Preparation of Minced Meat Gel

The samples were centrifuged at 5259× *g* for 5 min and then maintained at 70 °C for 30 min, followed by cooling at 4 °C for 12 h. The samples were taken out from the fridge and left at room temperature (22.0 ± 0.5 °C) for 30 min, resulting in the formation of minced meat gel.

### 2.5. Raman Spectroscopy

The minced meat was flattened on a clean glass slide and mounted on a carrier stage for Raman spectroscopy scanning (inVia, Renishaw plc, London, UK). The laser was focused on the processed minced meat with a 50× telephoto lens with a laser wavelength of 785 nm and a laser power of 100% (150 mW). Subsequently, the spectral acquisition conditions were established, comprising 10 scans, 1 s exposure time, and a resolution of 2 cm^−1^ in a wavenumber range of 300–2400 cm^−1^. The intensity at 1760 cm^−1^ was employed to illustrate the hydrophobic interaction of the minced lamb gel, whereas the I850/I830 values were utilized to indicate the hydrogen bonding of the minced lamb gel [20].

### 2.6. Fluorescence Scan

The minced meat samples were frozen at −18 °C for approximately 4 h and then lyophilized in a vacuum freeze-dryer (SCIENTZ-10ND, Ningbo Scientz Biotechnology Co., Ltd., Ningbo, China). Subsequently, the samples were ground into powder and stored in a desiccator at room temperature (22.0 ± 0.5 °C). The powder was dissolved in 0.01 mol/L PBS buffer (pH 7.0) to prepare a 0.2 mg/mL solution. The solution was recorded at 270 nm (SYNERGYH1, BioTek Instruments, Inc., Winooski, VT, USA), and the emission spectra were recorded from 300 to 450 nm with a constant slit width of 5 nm [21].

### 2.7. Differential Scanning Calorimetry (DSC)

The powder samples from Section 2.6 above (5 mg) were sealed in an aluminum pan and maintained at 20 °C for 2 min, followed by a gradual increase in temperature at a rate of 10 °C/min to 250 °C. An anhydrous, airtight, hermetically sealed aluminum dish was employed as a reference. The maximum transition temperature (Tmax) was determined using the universal analysis software (TA Universal Analysis 2000) provided by the DSC (DSC25, TA Instruments, New Castle, DE, USA) [22].

### 2.8. Low Field Nuclear Magnetic Resonance (LF-NMR) Measurement

The magnetic resonance imaging (MRI) analyzer (NMI20-060H-I, Suzhou Niumag Analytical Instrument Corporation, Suzhou, China) was operated at a proton resonance frequency of 23.4 MHz. A 10 g sample was weighed in a 15 mm glass tube, and the NMR probe was inserted into the tube. The transverse relaxation time T2 was evaluated using the Carr–Purcell–Meiboom–Gill (CPMG) sequence. T values were quantified using τ values (time between 90 and 180 pulses) obtained from 18,000 echoes as a result of eight scanning repetitions. The CPMG decay curves were subjected to distributed exponential fitting using a multiexponential fit analysis method, namely MultiExp Inv Analysis software (Mev 4.9). To ensure the accuracy of the results, it was essential to thermoregulate the heated minced meat samples at 70 °C for 30 min prior to testing [23].

### 2.9. Protein Properties

#### 2.9.1. Protein Solubility

Total protein solubility: A 1 g sample was weighed and dissolved in a suspension of 20 mL of 0.1 M potassium phosphate buffer (pH 7.2, 1.1 M KI). The phosphate buffer (pH 7.2) was homogenized for 3 × 20 s at a speed of 6500 rpm under ice bath conditions, with the temperature maintained at 4 °C. Subsequently, the slurry was subjected to centrifugation at 1500× *g* for 20 min at 4 °C. Soluble myoplasmic protein solubility: A 1g sample was weighed and dissolved in a suspension of 10 mL of 0.025 M potassium phosphate buffer (pH 7.2, 1.1 M KI). The phosphate buffer (pH 7.2) was homogenized for 2 × 20 s at a speed of 6500 rpm under ice bath conditions, with the temperature maintained at 4 °C. Subsequently, the slurry was subjected to centrifugation at 1500× *g* for 20 min at 4 °C. The concentration of proteins in the supernatant was determined by the Biuret method, allowing the solubility of total proteins and myoplasmic proteins to be calculated. The extent of proteolysis was expressed by the difference between total proteolysis and myoplasmic proteolytic solubility [24].

#### 2.9.2. Emulsion Stability

Total juice loss is a composite of water loss and fat loss. The samples were weighed (*M*_1_) into pre-weighed 50 mL centrifuge tubes (*M*_0_) and subjected to centrifugation at 5259× *g* for 5 min. Following this, the tubes were sealed with a cap. Subsequently, the tubes were placed in a water bath (HH-6, Changzhou Guohua Electric Appliances Co., Ltd., Changzhou, China) at 70 °C for 30 min, followed by pouring into a weighed Petri dish (*M*_2_). The samples were then left to stand for 1 h. The total weight of the tube and the sample was recorded (*M*_3_), and the collected liquid was heated to 103 °C for 16 h [25]. The total weight of the heated liquid was recorded (*M*_4_). The percentages of water, fat, and total juice loss were calculated using the following Equations (1)–(3):

(1) $$Percentage\;of\;total\;juice\;loss\left(\%\right)=\frac{\left(M_{0}{+M}_{1}\right)-M_{3}}{M_{1}}\times 100\%$$

(2) $$Percentage\;of\;water\;loss\left(\%\right)=\frac{{(M}_{1}+M_{0}-M_{3})-(M_{4}{-M}_{2})}{M_{1}}\times 100\%$$

(3) $$Percentage\;of\;fat\;loss\left(\%\right)=\frac{M_{4}-M_{2}}{\left(M_{0}{+M}_{1}\right)-M_{3}}\times 100\%$$

#### 2.9.3. Surface Hydrophobicity

The sample (0.6 g) was weighed and then dissolved in 20 mL of 0.02 M PBS buffer (pH 6.0) in order to form a suspension. The mixture was homogenized (XHF-DY, Ningbo Scientz Biotechnology Co., Ltd, Ningbo, China) at 7000 rpm for a period of 0.5 min. Subsequently, 100 μL of 1 mg/mL bromophenol blue was added to 1 mL of PBS buffer as a reference point. The mixture was shaken at 400 rpm for 20 min in a shaker followed by centrifugation at 1886× *g* for 15 min. The resulting supernatant was mixed with 4.5 mL of PBS buffer, and the absorbance value at 595 nm (*A*_2_) was measured using a spectrophotometer. The PBS buffer was used as a blank (*A*_1_) [26]. The surface hydrophobicity was calculated using the following Equation (4):

(4) $$Bromopheno\;blue\;binding/\mu g=100\;\mu g\frac{A_{1}-A_{2}}{A_{1}}$$

### 2.10. Gel Properties

#### 2.10.1. Microstructure Analysis

The minced lamb gel was poured into a 1 cm × 1 cm × 1 cm square container, and the container was immersed into 2.5% glutaraldehyde at 4 °C for 24 h to facilitate fixation. The samples were then rinsed with 0.01 mol/L PBS (pH 7.4) for 10 min at room temperature (22.0 ± 0.5 °C). This process was repeated three times, and subsequently immersed in 60%. Following this, the samples were soaked in 60%, 70%, 80%, 90%, and 100% ethanol solutions for 20 min, to achieve a further reduction in moisture content. Finally, the samples were immersed in tert-butanol for 10 min, on three separate occasions. Subsequently, the samples were dried with liquid nitrogen and affixed to the sample stage with conductive double-sided adhesive tape. They were then placed under the scanning electron microscope (SEM) (S-530, Hitachi, Ltd., Tokyo, Japan) for observation with the accelerating voltage set to 25 kV [27].

#### 2.10.2. Determination of Textural Properties and Gel Strength

The gel of minced lamb was formed into a cylinder with a radius of 25 mm and a height of 25 mm. The texture analyzer (TA.XT.Plus, Stable Micro Systems Ltd., Godalming, UK) was configured to TPA mode using a probe model P/36R with a strain ratio of 50%, a pre-trigger velocity of 5 mm/s, a mid-trigger velocity of 10 mm/s, and a post-trigger velocity of 15 mm/s. The return velocity was set to 5 mm/s, and the trigger force was set to 5 g. To determine the gel strength, the measurement mode was set to penetration mode, and the probe model P/0.5 was used, with a penetration ratio of 50% and a measuring speed of 1 mm/s [28].

#### 2.10.3. Cooking Loss

A quantity of 5 g of minced meat (*W*_0_) was placed into a pre-weighed centrifuge tube (*W*_1_), followed by cooking in a water bath at 85 °C for 15 min until the center temperature reached 75 °C, which was measured using a digital thermometer. Subsequently, the water was removed with filter paper, and the sample was reweighed (*W*_2_). For each treatment group, the measurements were carried out five times [29]. The steaming loss was calculated using the following Equation (5):

(5) $$Cooking\;loss:\frac{W_{1}-W_{2}}{W_{0}}\times 100\%$$

### 2.11. Statistical Analysis

The statistical data were presented in the form of the mean ± standard deviation (X ± SD). The test data were subjected to a one-way analysis of variance (ANOVA), and post hoc multiple comparisons Duncan analysis was performed using SPSS Statistics 25.0 statistical software, with a significance level of *p* < 0.05, to ascertain their statistical significance.

## 3. Results and Discussion

### 3.1. Raman Spectral Analysis

**Figure 2:**
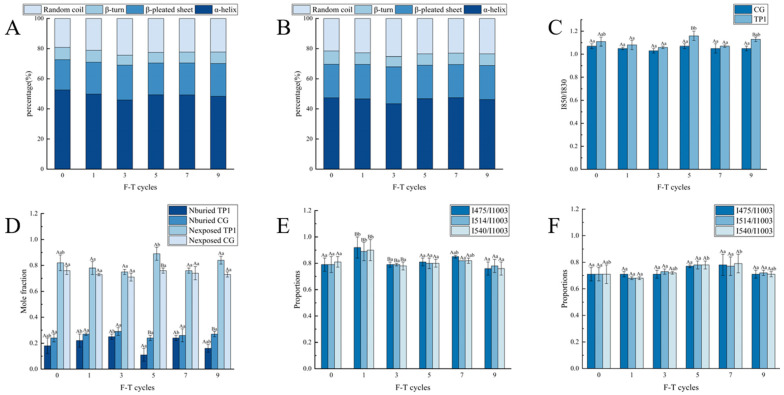
α-helices, β-pleated sheet, β-turn, and random coil of the minced lamb protein under six freeze–thaw conditions: (**A**) CG; (**B**) TP1; (**C**) changes in the number of CG and TP1 hydrogen bonds under six freeze–thaw conditions; (**D**) changes in the hydrogen bonding of different conformations of CG and TP1 under six freeze–thaw conditions; changes in the number of disulfide bonds under six freeze–thaw conditions; (**E**) CG; (**F**) TP1. Different shoulder-marked lowercase letters in the same column indicate significant differences between groups (*p* < 0.05). Capital letters with different shoulder labels indicate significant differences within groups (*p* < 0.05).

As shown in Figure 2A,B, the proportions of α-helices in all treatment groups exhibited a significant declining trend during the first three freeze–thaw cycles (*p* < 0.05). Compared to their non-freeze–thawed states, the α-helix proportions in CG and TP1 groups decreased by 7.21% and 4.00%, respectively. The alteration in the proportion of random coils exhibits an inverse correlation to that of the proportion of α-helices. This is due to the fact that the stability of the α-helix structure depends on the hydrogen bonds between the carbonyl oxygen and the amino hydrogen in the polypeptide chain [30]. The formation of ice crystals and the redistribution of water molecules during repeated freeze–thaw cycles caused alterations in the physical and protein structure, and consequently a denaturation of proteins due to the broken hydrogen bonds. The loss in hydrogen bonds resulted in the transformation of α-helixes from an ordered state to an irregularly coiled state [31]. The addition of tea polyphenols (TPs) showed an increase in β-pleated sheets, β-turns, and random coils (Table S1), but the difference was not significant (*p* > 0.05). Parallel studies have also identified inconsistencies in the impact of phenolic compounds on protein secondary structures. These discrepancies may be attributed to the structural flexibility, molecular weight, and OH groups of the polyphenols, as well as the proteins themselves [32].

The tyrosine residue microenvironment was monitored via bimodal band vibrations at 830 and 850 cm^−1^. The 850 cm^−1^ peak corresponds to the in-plane C-C/C-O deformation vibration of the tyrosine benzene ring, which dominates and exhibits high intensity when the phenolic hydroxyl group remains non-hydrogen bonded. Conversely, when the phenolic hydroxyl forms a hydrogen bond with other groups (e.g., intra-protein polar groups or water molecules), the benzene ring’s electron cloud distribution is altered, leading to a redshift of this vibrational mode to 830 cm^−1^ and a concomitant increase in peak intensity. The electrostatic effect of hydrogen bonding reduces the benzene ring’s symmetry, causing the originally degenerate vibrational mode to split, thereby modifying the intensities of the 830 cm^−1^ and 850 cm^−1^ peaks. An I850/I830 ratio < 1.0 indicates hydrogen bonding, whereas a ratio > 1.0 signifies the absence of hydrogen bonding [33]. The microenvironment is susceptible to alteration by external factors, as well as the participation of phenolic hydroxyl groups in hydrogen bonding. The alterations in hydrogen bonding in freeze–thawed lamb mince are illustrated in Figure 2C. No significant changes were observed in the number of hydrogen bonds among all treatment groups (*p* > 0.05). It is important to note that the hydrogen bond between the carbonyl group and the amino group plays a crucial role in stabilizing the α-helix structure. The reduction in the number of tyrosine phenolic hydroxyl groups was correlated with a corresponding reduction in the proportion of α-helices. This may have contributed to the deterioration of the water retention capacity of minced lamb after the third freeze–thaw cycle. As illustrated in Figure 2D, TP1 exhibited a higher number of hydrogen bonds at all freeze–thaw cycles when compared to CG. Meanwhile, the number of Nburied in TP1 was found to be lower than that in CG, with a significant difference (*p* < 0.05) at the ninth freeze–thaw cycle (Table S2). This suggests that TPs preserve the effect of maintaining the numbers of hydrogen bonds during the freeze–thaw process. Concurrently, as the proteins are cross-linked with TPs, forming hydrogen bonds, a proportion of the -OH groups on the TPs molecules may also form hydrogen bonds with water molecules, thereby enhancing the hydrogen bonding between proteins. This may explain why the addition of a moderate amount of TPs resulted in enhanced emulsion stability and gelation strength in minced lamb [34].

The alterations in the hydrophobic interactions of lamb mince are illustrated in Figure S1 [35]. The surface hydrophobicity of CG exhibited a notable increase after a single freeze–thaw treatment (*p* < 0.05). However, the hydrophobicity of TP1 was lower than that of CG. This may be due to the reduced surface hydrophobicity of minced lamb. Tea polyphenols, as antioxidants, contain a 2-phenylbenzopyran structure. When combined with hydroxyl groups, this structure exhibits strong free radical scavenging ability, inhibiting protein oxidative denaturation. This inhibition leads to a reduction in the exposure of hydrophobic groups on the protein surface, thereby decreasing protein hydrophobicity [36].

The vibrations in the range of 460–679 cm^−1^ are indicative of the presence of disulfide and sulfhydryl (SH) bonds in three distinct conformations of the protein. Of these bonds, the disulfide bond plays a pivotal role in maintaining protein conformation [20]. As shown in Figure 2E, F, the number of disulfide bonds in the different conformations of CG was significantly higher (*p* < 0.05) after one freeze–thaw treatment, with values of 0.92, 0.89, and 0.90 (Table S3), respectively. This stems from protein denaturation/folding during freeze–thaw cycles and the oxidation of amino acid sulfhydryl groups, driving SH-to-disulfide bond conversion [37,38]. The number of disulfide bonds in TP1 was significantly lower than that in CG (*p* < 0.05) after a single freeze–thaw cycle. A considerable number of phenolic hydroxyl groups in TPs molecules may inhibit both intermolecular and intramolecular rearrangements of proteins by binding with hydrophobic groups and slowing down the rate of oxidative protein denaturation. Following the scavenging of free radicals, TPs form semiquinone radical structures or quinone compounds [39]. This process inhibited the formation of disulfide bonds. Thus, the gel strength of minced lamb gel was significantly reduced with the addition of TPs.

### 3.2. Fluorescence Spectral Analysis

**Figure 3:**
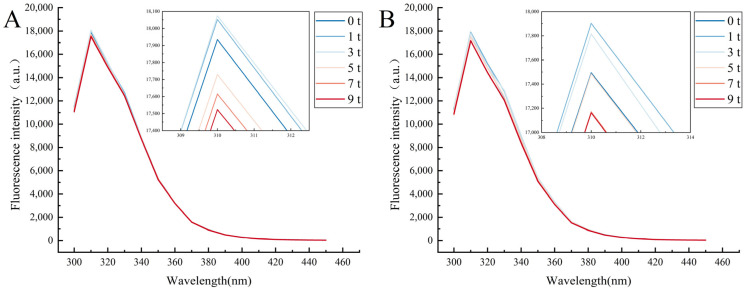
Fluorescence profiles of (**A**) CG and (**B**) TP1 at 300~450 nm.

Proteins containing aromatic amino acids, such as tryptophan, tyrosine, and phenylalanine, fluoresce under certain excitations, called intrinsic protein fluorescence. Among these amino acids, the fluorescence of tryptophan is typically the most prominent, and tryptophan is considerably more sensitive to its surrounding environment than other amino acids. Consequently, protein tryptophan fluorescence can provide crucial insights into protein structure [40]. As shown in Figure 3, when the excitation wavelength was set at 270 nm, the maximum fluorescence emission wavelength of CG did not change significantly (*p* > 0.05). However, there was a notable decline in fluorescence intensity with an increase in the number of freeze–thaw cycles. The repeated freezing and thawing of the protein resulted in aggregation, which increased the spatial steric hindrance and led to a decrease in fluorescence intensity [41]. The fluorescence intensity of TP1 was remarkably diminished in comparison to that of CG under non-freeze–thaw conditions, indicating that the addition of tea polyphenols (TPs) altered the protein structure, leading to the loss of intrinsic fluorescence. It was proposed that water-soluble phenolic compounds could decrease the overall fluorescence of myofibrillar proteins by enhancing the polar microenvironment of myofibrillar amino acid residues and providing a shielding effect on nonpolar residues. In this context, the addition of TPs to myofibrillar proteins altered the microenvironment of tryptophan residues from being hydrophobic to hydrophilic, indicating that the protein became less hydrophobic when interacting with TPs [42,43].

### 3.3. Differential Scanning Calorimetry (DSC) Analysis

**Figure 4:**
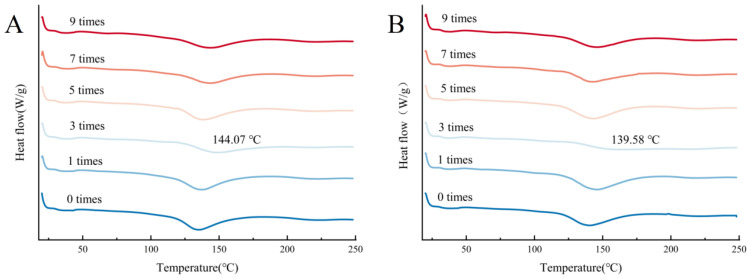
Change curves of DSC in (**A**) CG and (**B**) TP1.

In Figure 4, the temperature dependence of the DSC curve of minced lamb exhibited an absorption peak at 120–150 °C, which indicates the denaturation temperatures of proteins under different treatments. After three freeze–thaw cycles, the thermal denaturation temperatures of proteins in both CG and TP1 groups increased significantly (*p* < 0.05) to 144.07 °C and 139.58 °C, respectively (Table S4). The freezing treatment has caused the protein to elongate and unfold, while the thawing process resulted in the refolding of unfolded protein molecular chains. To some extent, the restoration of the protein resulted in a change in the thermal denaturation temperature [44]. The protein thermal denaturation temperature of TP1 was reduced compared to CG, which may reflect that the change in thermal denaturation temperature may be caused by the change in the protein side chain motifs as tea polyphenols bind to proteins through covalent and non-covalent bindings, which leads to the disruption of hydrophobic interactions. The altered thermal transitions result from the modification of protein side chain moieties, and may also stem from cross-linking or interaction with TPs (polar and charged groups), which disrupt the intramolecular electrostatic forces within myosin [45]. Collectively, the addition of TPs decreased the thermal stability of proteins [46].

### 3.4. Low Field Nuclear Magnetic Resonance (LF-NMR) Analysis

**Figure 5:**
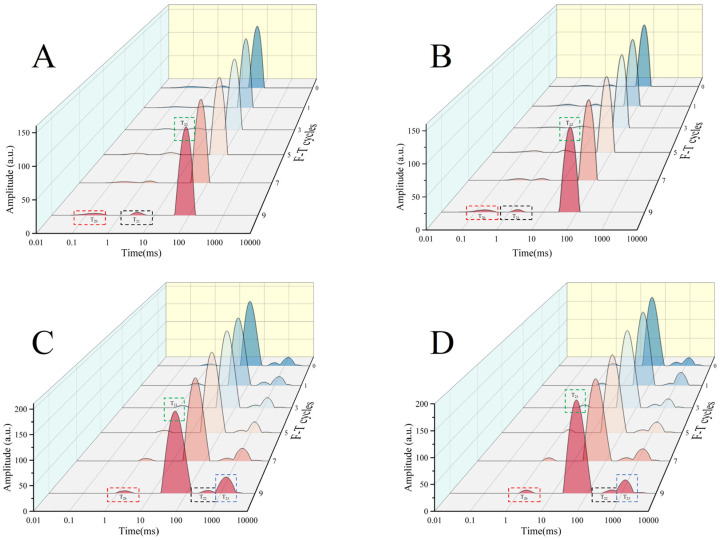
Plot of peak transverse relaxation time of minced lamb for the six freeze–thaw conditions: (**A**) CG before steaming, (**B**) TP1 before steaming, (**C**) CG after steaming, and (**D**) TP1 after steaming. Different colored dashed frames represent different types of bound water. The red dashed frame represents strongly bound water, the black dashed frame represents weakly bound water, and the green dashed frame represents not readily mobile water (**A**,**B**). The red dashed frame represents strongly bound water, the green dashed frame represents weakly bound water, and the black dashed frame and the blue dashed frame represent not readily mobile water (**C**,**D**).

Figure 5 presents the results from LF-NMR, which reflect the degree of water freedom by the magnitude of T2 relaxation time for minced lamb. As shown in Figure 5A, the observed three peaks, from left to right, represent strongly bound water (T_2b_: 0.1–1 ms), weakly bound water (T_21_: 1–10 ms), and not readily mobile water (T_22_: 10–100 ms), respectively. The peak at T_22_ was the most prominent among all treatment groups, demonstrating that the predominant form of water in the minced lamb was not readily mobile water. Therefore, the freezing and thawing processes, as well as the addition of TPs, did not exert a notable influence on the water form in the minced lamb (Figure 5B). T_2b_, T_21_, and T_22_ of all treatment groups demonstrated an increase from the first to the third freeze–thaw cycle, followed by a decline from the third to fifth cycle (Table 1). As the freeze–thaw cycle progressed, water molecules were extruded from the interior of myofibers to the exterior of the cell. This enhanced the ability of myofibers to reabsorb water molecules, consequently decreasing the relaxation time [47]. The T_2b_ of TP1 was significantly greater than that of CG at 0 and 1 freeze–thaw cycles. This was primarily due to the addition of TPs, which altered the interactions among proteins as well as between proteins and water molecules, thus increasing the degree of freedom of water molecules. Following the seventh freeze–thaw cycle, the T_2b_ of CG was found to be significantly higher than that of TP1 (*p* < 0.05). This may be attributed to the fact that, following the interactions with proteins, TP1 was able to absorb a considerable number of water molecules through hydrogen bonding, thereby enhancing the protein gel’s water-binding capacity and maintain hydration. The oxidation of polyphenols generates quinone intermediates, which form covalent bonds with nucleophilic residues (e.g., amino groups of lysine, thiol groups of cysteine) in protein side chains. This cross-linking modifies protein surface charge and spatial conformation, thereby influencing hydration behavior. Specifically, it enhances protein network density, reduces water exudation, and improves water-holding capacity [48]. In contrast, CG exhibited a relatively high degree of oxidation, which altered the structure of the proteins and their side chain groups, reducing the tightness of the bond between them and water molecules, thereby resulting in an increase in the relaxation time [49]. Furthermore, the peak area of T_2b_ and T_22_ did not change significantly with increasing freeze–thaw cycles (*p* > 0.05) (Table S5), indicating that the water molecules in T_2b_ are tightly bound to proteins and other macromolecules, while the water molecules in T_22_ were encapsulated inside the myofibrils and were more tightly bound [50].

During the steaming process of minced lamb, protein molecules were denatured and transformed into gels under the influence of heat. As shown in Figure 5C,D, a new peak representing not readily mobile water appeared (T_23_: > 1000 ms). This peak may be attributed to the gradual formation of proteins in minced lamb into a gel during the cooking process, following heat denaturation and swelling. During this process, some water molecules were absorbed, while some water molecules were extruded. T_2b_ in CG increased with the increase in number of freeze–thaw cycles, indicating the migration of bound water to free water (Table 2). This may be attributed to the alterations in the secondary and tertiary structures of proteins caused by freezing and thawing, which consequently reduced their binding capacity to water molecules [51]. It is shown that the relaxation time of TP1 has significantly decreased (Table S6) [52,53], which may be due to the strong binding of water molecules to the protein gel, resulting in a more stable and less porous gel system due to the incorporation of TP molecules. Following cross-linking with proteins, TPs might absorb a significant number of water molecules through hydrogen bonding, thereby enhancing the capacity of protein gels to bind to water and maintain hydration. Furthermore, the interactions between TPs and proteins have been shown to reduce the gaps within the protein polymer chains to a certain extent, thereby making the network denser, restricting the movement of ^1^H atoms, and decreasing the mobility of water molecules [54]. Meanwhile, Table S7 demonstrated the changes in the peak area ratio of the T2 relaxation time of minced meat after steaming, in which P_23_ of TP1 was significantly lower than that of CG, which indicated that the addition of TPs reduced the loss of free water and enhanced the hydration capacity of proteins.

Comparative analysis of the relaxation time of unheated minced lamb with that of minced lamb after heating revealed a significant increase in the latter. It is believed that heating may mobilize a portion of water and fat in the minced lamb within the gel. This finding suggests that proteins in a solubilized state may possess superior water and fat binding capabilities compared to their state after gel formation [55].

### 3.5. Changes in the Nature of Proteins in Minced Lamb

#### 3.5.1. Changes in Protein Solubility of Minced Lamb

**Figure 6:**
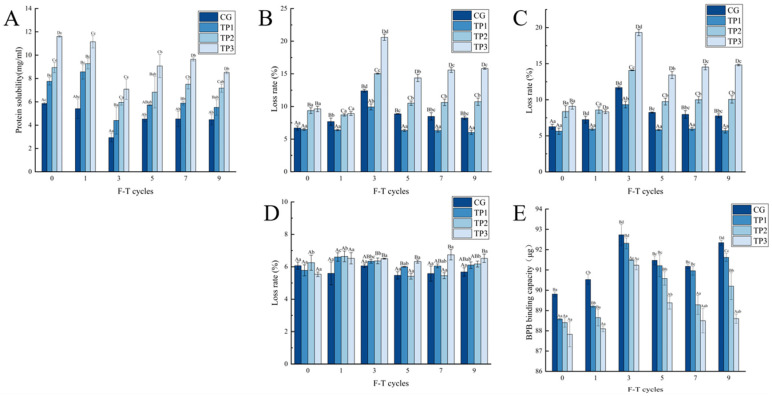
(**A**) Protein solubility of minced lamb with different amounts of TPs under six freeze–thaw conditions; loss rate of minced lamb with different tea polyphenol additions under different freeze–thaw conditions: (**B**) Total loss; (**C**) water loss; (**D**) fat loss; (**E**) changes in surface hydrophobicity of minced lamb with different levels of TPs under different freeze–thaw conditions. Different shoulder-marked lowercase letters in the same column indicate significant differences between groups (*p* < 0.05). Capital letters with different shoulder labels indicate significant differences within groups (*p* < 0.05).

The variation in protein solubility in minced lamb with the increasing number of freeze–thawing cycles is presented in Figure 6A. The protein solubility in all treatment groups exhibited a decreasing trend during 1–3 cycles of freeze–thawing, followed by an increasing trend during 3–5 cycles of freeze–thawing. The protein solubility demonstrated a nadir at the third cycle, with values recorded at 2.93 mg/mL, 4.40 mg/mL, 5.96 mg/mL, and 7.08 mg/mL (Table S7), respectively. The results indicated that the structure of protein was altered during the freeze–thaw cycles. This caused the exposure of the hydrophobic groups to the surface, triggering coagulated and precipitated of protein molecules, which ultimately led to a decreased myofiber proteolysis [56,57]. The carbonyl content of myofibrillar proteins showed a continuous increase after five freeze–thaw cycles, accompanied by the degradation of myosin heavy chains. This indicates a transition from protein cross-linking to fragmentation, releasing partially soluble proteins and thus increasing protein solubility [58]. Under the same freeze–thaw conditions, protein solubility with TPs demonstrated an increasing trend with the level of TP concentration. This phenomenon could be attributed to the ability of TPs to reduce protein oxidation and polymerization [59]. It has been reported that phenolic hydroxyl groups could enhance the hydrophilicity of protein surfaces, thereby increasing protein solubility due to the reduction in protein hydrophobicity and the increase in protein charges [60].

#### 3.5.2. Changes in the Emulsion Stability of Minced Lamb

As shown in Figure 6B–D, the emulsion stability of lamb mince was found to be dependent on the number of freeze–thaw cycles. The total juice loss rate, water loss rate, and fat loss rate of all treatment groups increased during 1–3 cycles of freeze–thaw cycles and a decreased trend during 3–9 cycles of freeze–thaw cycles (*p* < 0.05). The repetitive freeze–thaw cycles induced changes in protein structure, thus hindering the formation of stable interfacial interactions. Furthermore, the undesirable aggregation of myosin may result in the excessively large protein molecules adsorbed on the surface of oil droplets [61]. During the 1–9 freeze–thaw cycles, the three loss rates (juice loss rate, water loss rate, and fat loss rate) of TP1 were significantly lower than those of CG (*p* < 0.05), attributed to the interactions between the phenolic hydroxyl groups in TPs with the protein residues. This interaction led to a stronger protein–fat interface, thereby enhancing the stability of the minced lamb system. On the contrary, the one to nine freeze–thaw cycles led to significantly higher loss rates of TP2 and TP3 than those of CG (*p* < 0.05) due to the excessive amount of TPs which interrupted the protein–fat interface. As the content of tea polyphenols increases, the interfacial adsorption sites become saturated, and polyphenols start to react with proteins to form aggregates. Consequently, oils and fats cannot be stably adsorbed on the protein surface, leading to emulsion instability [62].

#### 3.5.3. Changes in Surface Hydrophobicity

As shown in Figure 6E, the surface hydrophobicity of all treatment groups increased during 0–3 rounds of freeze–thaw cycles and decreased during 3–5 rounds of freeze–thaw cycles. The surface hydrophobicity reached its maximum value at the third freeze–thaw cycle for all groups at 92.73 mg, 92.31 mg, 91.49 mg, and 91.23 mg, respectively. The alterations in the protein structure induced by the freeze–thaw treatment caused the exposure of hydrophobic groups, which were originally embedded within the protein’s interior side, to the surface. This increased the surface hydrophobicity of the minced lamb observed at the third freeze–thaw cycle. The surface hydrophobicity exhibited a downward trend in response to the increased amount of TP additions. The hydrophobicity of TP3 was significantly lower than that of CG except at day treatment (*p* < 0.05). This may have been due to the ability of TPs to suppress the oxidative denaturation of proteins in minced lamb while reducing the number of hydrophobic aliphatic and aromatic groups revealed internally. Furthermore, the hydrophilic groups introduced by the TPs contributed to a reduction in surface hydrophobicity [36]. The decrease in hydrophobicity observed in the presence of high doses of TPs can be attributed to the aggregation of myofibrillar proteins, which results in the continuous exposure of hydrophobic groups. This, in turn, leads to hydrophobic interactions among the proteins, thereby blocking the hydrophobic moieties and rendering them inaccessible to bromophenol blue [63].

### 3.6. Changes in Gel Properties of Minced Lamb

#### 3.6.1. Changes in Gel Microstructure

**Figure 7:**
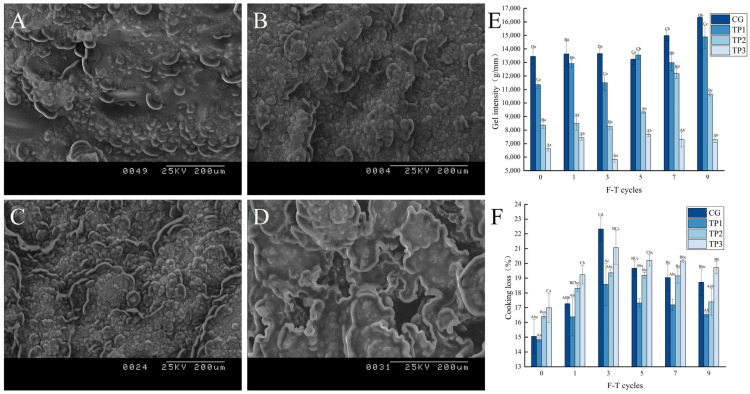
Gel microscopic images of minced lamb at the third freeze–thaw: (**A**) CG; (**B**) TP1; (**C**) TP2; (**D**) TP3; (**E**) gel strength of minced lamb with different amounts of TPs added under six freeze–thaw conditions; (**F**) cooking loss of minced lamb with different amounts of TPs added under six freeze–thaw conditions. Different shoulder-marked lowercase letters in the same column indicate significant differences between groups (*p* < 0.05). Capital letters with different shoulder labels indicate significant differences within groups (*p* < 0.05).

Figure 7 presents the gel micromorphology of minced lamb with varying amounts of TPs during three freeze–thaw cycles. The distribution of the gel microstructure in CG (Figure 7A) was found to be more uniform in comparison to that observed in TP1 (Figure 7B). This difference is primarily attributed to phenol–protein interactions, especially covalent cross-linking. As the bonding between polyphenols and proteins increases, micelles form, resulting in a more compact structural arrangement [64]. With the increase in TPs, the gel structure became more rough and uneven, and more irregular pores and larger pore sizes appeared. It was reported that the oxidation products of TPs may react with sulfhydryl groups and inhibit the formation of disulfide bonds, which impedes the formation of an orderly minced meat gel network [65].

#### 3.6.2. Changes in Gel Textural Properties

In Table 3, the gel strength of minced lamb varied with the number of freeze–thaw cycles. It is evident that the hardness of all treatment groups exhibited its maximum value at the third freeze–thaw cycle, with the values of 11,109.69 g, 10,618.35 g, 10,063.29 g, and 6026.25 g, respectively. It indicated that the repeated freeze–thaw cycles resulted in the damage of muscle tissues and cell membranes of minced lamb, leading to an irreversible change in proteins, which, in turn, resulted in inadequate water retention and a hard texture. Under all freeze–thaw conditions, the hardness, cohesiveness, chewiness, and responsiveness of TP1 were significantly lower than those of CG (*p* < 0.05). In addition, these parameters increased with the increase in TP concentration. The decrease in gel strength at high tea polyphenol concentrations can be attributed to excessive polyphenol–protein interactions, which hinder protein–protein interactions during thermal gelation and thereby impair gel strength [66]. Epigallocatechin gallate (EGCG) is an important functional substance in TPs. It was discovered that the addition of a high concentration of EGCG led to a decrease in the number of sulfhydryl groups and free amino groups in proteins, thereby inhibiting the cross-linking effect in the formation of heat-induced gel. This, in turn, resulted in a loosened organizational structure of the system and a deterioration of its qualitative and structural properties [46]. This may have contributed to the deterioration of the gelatinization properties of minced lamb.

#### 3.6.3. Changes in Gel Strength

As shown in Figure 7E, the gel strength of minced lamb varied significantly. A notable absence of a substantial correlation was observed between gel strength and the number of freeze–thaw cycles across the four treatment groups. However, in all freeze–thaw cycles, a significant decrease in gel strength occurred with the increase in TP addition (*p* < 0.05). The gel strength of TP3 had a minimum value of 5826.29 g/mm at the third freeze–thaw cycle. At suitable levels of polyphenol content, the oxidation of polyphenols resulted in the formation of quinones which, subsequently, promoted the conversion of sulfhydryl groups in myofibrillar proteins to disulfide bonds by acting as a cross-linking agent by covalently binding to various nucleophilic groups to form an organized gel network [67]. On the contrary, high doses of TPs inhibited gel cross-linking through OH attack, which led to the formation of excess quinones. These quinones may have then reacted with the -SH or -NH2 groups of proteins to form quinone-thiol or quinone-amino adducts, respectively [68]. These adducts then bind to another protein to form a polymer with a high site barrier, which causes the gel strength to be reduced.

#### 3.6.4. Changes in Cooking Loss

As shown in Figure 7F, the cooking loss of minced lamb varied remarkably. The cooking loss of all treatment groups demonstrated an increase during 1–3 rounds of freeze–thaw cycles, and a decreasing trend during 3–9 rounds of freeze–thaw cycles. The maximum cooking loss was at the third freeze–thaw cycle, with an increase of 7.27%, 3.74%, 2.95%, and 4.08%, respectively, compared to the initial state. This result showed the damage to the protein structure by ice crystals during the freeze–thaw cycles; as a result, this affected the formation of their gel network and led to a decrease in the water-holding capacity [69]. The cooking loss of TP1 was significantly lower than that of CG (*p* < 0.05), suggesting that small amounts of TPs could improve the formation of protein gels and enhance their stability. This may be due to the interaction between polyphenol hydroxyl groups and protein disulfide and non-disulfide bonds to form a more stable structure [70]. The results of this study were consistent with those of previous studies, which indicated that phenolic compounds were advantageous to the water-holding capacity of muscle emulsions by reducing myosin heavy chain polymerization and retaining myosin heavy chain thiols. However, it should be noted that the excessive TPs might lead to an increase in cooking loss. Figure 7 also showed that the cooking loss of TP2 and TP3 were significantly higher than those of TP1 (*p* < 0.05) due to the excessive TPs which resulted in the occurrence of “mercapto-quinone” and “amino-quinone” reactions. These reactions have the potential to induce undesirable cross-linking or aggregation, thereby occluding part of the reactive groups. Furthermore, due to spatial and positional constraints, additional interactions are impeded, consequently resulting in an escalation in the cooking loss [71].

## 4. Conclusions

Repeated freeze–thaw cycles degrade minced lamb proteins, impairing emulsification and gel properties. Tea polyphenols (TPs) mitigate this damage by reducing protein oxidation, juice loss, and surface hydrophobicity. Low-dose TP supplementation (0.01%) enhances gel texture, preserving meat quality during freezing–thawing. Notably, TPs exhibit dual effects: while protecting against freeze–thaw injury, higher concentrations may induce protein aggregation. This highlights the need to optimize TP dosage for balanced antioxidant benefits and minimal side effects. Mechanistic insights establish TPs as a viable natural preservative, with future research needed to explore synergistic strategies for improved meat stability.

## Figures and Tables

**Table 1:** Changes in transverse relaxation time of minced lamb after freeze–thaw cycles before cooking treated with tea polyphenols.

F-T Cycles	Groups	T_2b_/ms	T_21_/ms	T_22_/ms
0	CG	0.17 ± 0.02 ^Aa^	2.20 ± 0.07 ^Aa^	60.84 ± 2.11 ^Aa^
	TP1	0.27 ± 0.03 ^Bb^	2.71 ± 0.09 ^Bab^	65.21 ± 2.26 ^Ba^
1	CG	0.18 ± 0.03 ^Aa^	3.19 ± 0.70 ^Ab^	72.50 ± 5.03 ^Ab^
	TP1	0.33 ± 0.02 ^Bc^	3.47 ± 0.63 ^Ab^	75.29 ± 7.81 ^Ab^
3	CG	0.26 ± 0.03 ^Ab^	2.79 ± 0.32 ^Aab^	70.24 ± 7.29 ^Aab^
	TP1	0.26 ± 0.01 ^Ab^	2.84 ± 0.25 ^Aab^	68.13 ± 9.40 ^Aab^
5	CG	0.22 ± 0.01 ^Aab^	2.31 ± 0.15 ^Aab^	67.64 ± 4.69 ^Aab^
	TP1	0.25 ± 0.02 ^Ab^	2.64 ± 0.09 ^Bab^	67.64 ± 4.69 ^Aab^
7	CG	0.26 ± 0.00 ^Bb^	2.20 ± 0.48 ^Aa^	64.08 ± 1.96 ^Aab^
	TP1	0.18 ± 0.03 ^Aa^	2.87 ± 0.77 ^Aab^	62.95 ± 0.00 ^Aa^
9	CG	0.20 ± 0.04 ^Aab^	2.44 ± 0.41 ^Aab^	73.02 ± 10.07 ^Ab^
	TP1	0.19 ± 0.01 ^Aa^	2.06 ± 0.18 ^Aa^	62.95 ± 0.00 ^Aa^

Note: The results are expressed as the mean ± SD. Different shoulder-marked lowercase letters in the same column indicate significant differences between groups (*p* < 0.05). Capital letters with different shoulder labels indicate significant differences within groups (*p* < 0.05). The control group (CG) consisted of minced lamb without tea polyphenol addition, while group TP1 contained minced lamb supplemented with 0.01% tea polyphenols.

**Table 2:** Changes in transverse relaxation time of minced lamb after freeze–thaw cycles after cooking treated with tea polyphenols.

F-T Cycles	Groups	T_2b_/ms	T_21_/ms	T_22_/ms	T_23_/ms
0	CG	1.40 ± 0.19 ^Aa^	46.09 ± 1.60 ^Aa^	457.76 ± 47.50 ^Aa^	1431.46 ± 0.00 ^Ba^
	TP1	1.49 ± 0.10 ^Aa^	47.69 ± 0.00 ^Ab^	490.67 ± 50.91 ^Aa^	1335.45 ± 0.00 ^Aab^
1	CG	1.61 ± 0.49 ^Aa^	53.72 ± 9.23 ^Ab^	588.30 ± 178.04 ^Aa^	1589.52 ± 55.15 ^Ab^
	TP1	1.83 ± 0.13 ^Ab^	58.73 ± 0.00 ^Ac^	622.26 ± 0.00 ^Ab^	1703.80 ± 59.12 ^Ad^
3	CG	1.67 ± 0.29 ^Aa^	51.11 ± 0.00 ^Bab^	541.59 ± 0.00 ^Ba^	1431.46 ± 0.00 ^Aa^
	TP1	1.54 ± 0.05 ^Aa^	47.69 ± 0.00 ^Ab^	488.32 ± 16.94 ^Aa^	1383.46 ± 48.00 ^Ab^
5	CG	1.62 ± 0.33 ^Aa^	51.11 ± 0.00 ^Bab^	506.48 ± 35.11 ^Aa^	1383.46 ± 48.00 ^Aa^
	TP1	1.76 ± 0.06 ^Ab^	47.69 ± 0.00 ^Ab^	488.32 ± 16.94 ^Aa^	1482.91 ± 51.45 ^Ac^
7	CG	1.79 ± 0.31 ^Aa^	51.11 ± 0.00 ^Bab^	523.43 ± 18.16 ^Aa^	1431.46 ± 0.00 ^Aa^
	TP1	1.76 ± 0.06 ^Ab^	47.69 ± 0.00 ^Ab^	472.51 ± 32.75 ^Aa^	1383.46 ± 48.00 ^Ab^
9	CG	1.90 ± 0.20 ^Aa^	47.69 ± 0.00 ^Aab^	439.76 ± 0.00 ^Aa^	1431.46 ± 0.00 ^Ba^
	TP1	1.76 ± 0.06 ^Ab^	46.09 ± 1.60 ^Aa^	488.32 ± 16.94 ^Aa^	1290.67 ± 44.78 ^Aa^

Note: The results are expressed as the mean ± SD. The control group (CG) consisted of minced lamb without tea polyphenol addition, while group TP1 contained minced lamb supplemented with 0.01% tea polyphenols. Different shoulder-marked lowercase letters in the same column indicate significant differences between groups (*p* < 0.05). Capital letters with different shoulder labels indicate significant differences within groups (*p* < 0.05).

**Table 3:** Textural properties of minced lamb gels with different tea polyphenols additions under six freeze–thaw conditions.

Textural Property	F-T Cycles	CG	TP1	TP2	TP3
hardness/g	0	10,608.04 ± 518.54 ^Dbc^	9375.32 ± 121.02 ^Cabc^	8507.81 ± 496.25 ^Ba^	5551.66 ± 383.60 ^Ac^
	1	10,357.29 ± 957.30 ^Cbc^	9160.55 ± 324.84 ^Ba^	9155.21 ± 253.74 ^Bb^	5842.65 ± 302.10 ^Acd^
	3	11,109.69 ± 267.40 ^Dc^	10,618.35 ± 54.13 ^Cd^	10,063.29 ± 67.57 ^Bc^	6026.25 ± 95.95 ^Ad^
	5	9337.44 ± 167.32 ^Ca^	9784.76 ± 291.76 ^Cbc^	8412.92 ± 358.35 ^Ba^	4590.79 ± 143.41 ^Ab^
	7	10,516.25 ± 263.52 ^Dbc^	9820.79 ± 191.80 ^Cc^	8533.88 ± 193.97 ^Ba^	3513.73 ± 157.36 ^Aa^
	9	9722.12 ± 75.11 ^Cab^	9299.54 ± 428.30 ^Cab^	8290.09 ± 364.57 ^Ba^	3637.52 ± 117.60 ^Aa^
resilience	0	0.89 ± 0.01 ^Ba^	0.90 ± 0.02 ^Ba^	0.88 ± 0.00 ^Ba^	0.83 ± 0.01 ^Ad^
	1	0.90 ± 0.01 ^Bbc^	0.90 ± 0.03 ^Ba^	0.89 ± 0.00 ^Ba^	0.83 ± 0.00 ^Ad^
	3	0.92 ± 0.01 ^Cc^	0.91 ± 0.01 ^Ca^	0.89 ± 0.01 ^Ba^	0.75 ± 0.02 ^Ac^
	5	0.90 ± 0.02 ^Bbc^	0.91 ± 0.02 ^Ba^	0.87 ± 0.05 ^Ba^	0.71 ± 0.01 ^Ab^
	7	0.91 ± 0.02 ^Bbc^	0.91 ± 0.00 ^Ba^	0.87 ± 0.01 ^Ba^	0.70 ± 0.03 ^Ab^
	9	0.92 ± 0.01 ^Cc^	0.91 ± 0.02 ^Ca^	0.86 ± 0.01 ^Ba^	0.65 ± 0.01 ^Aa^
cohesiveness	0	0.69 ± 0.01 ^Cb^	0.73 ± 0.00 ^Dd^	0.68 ± 0.03 ^Bb^	0.61 ± 0.00 ^Ad^
	1	0.70 ± 0.00 ^Cb^	0.72 ± 0.00 ^Dc^	0.68 ± 0.00 ^Bb^	0.60 ± 0.00 ^Ad^
	3	0.69 ± 0.00 ^Cb^	0.72 ± 0.00 ^Dc^	0.65 ± 0.00 ^Ba^	0.56 ± 0.01 ^Ac^
	5	0.67 ± 0.01 ^B Ca^	0.70 ± 0.00 ^Cb^	0.64 ± 0.00 ^Ba^	0.51 ± 0.03 ^Ab^
	7	0.66 ± 0.00 ^Ca^	0.69 ± 0.00 ^Da^	0.64 ± 0.02 ^Ba^	0.48 ± 0.00 ^Aa^
	9	0.70 ± 0.00 ^Cb^	0.70 ± 0.00 ^Cb^	0.63 ± 0.00 ^Ba^	0.46 ± 0.00 ^Aa^
chewiness/g	0	6958.15 ± 42.03 ^Db^	6225.65 ± 54.88 ^Cb^	4914.27 ± 351.05 ^Bbc^	3090.02 ± 192.16 ^Ae^
	1	6977.87 ± 455.21 ^Db^	5929.07 ± 190.35 ^Cab^	5158.12 ± 382.81 ^Bc^	2822.79 ± 18.19 ^Ad^
	3	6240.97 ± 676.08 ^Ca^	5746.30 ± 22.08 ^B Ca^	5164.81 ± 110.80 ^Bc^	2269.00 ± 114.38 ^Ac^
	5	6140.44 ± 197.18 ^Ca^	6030.01 ± 193.83 ^Cab^	4529.69 ± 81.73 ^Bab^	1562.00 ± 65.53 ^Ab^
	7	5820.34 ± 190.08 ^Ca^	5843.48 ± 266.69 ^Ca^	4872.54 ± 222.29 ^Bbc^	1631.54 ± 101.57 ^Ab^
	9	6093.82 ± 147.47 ^Ca^	5855.64 ± 197.45 ^Ca^	4332.49 ± 37.24 ^Ba^	1089.77 ± 56.10 ^Aa^
responsiveness	0	0.29 ± 0.00 ^Dc^	0.33 ± 0.00 ^Cb^	0.24 ± 0.01 ^Ba^	0.21 ± 0.00 ^Ad^
	1	0.32 ± 0.01 ^Cd^	0.34 ± 0.01 ^Db^	0.28 ± 0.00 ^Bc^	0.22 ± 0.00 ^Ad^
	3	0.29 ± 0.00 ^Cbc^	0.33 ± 0.00 ^Db^	0.25 ± 0.00 ^Bab^	0.19 ± 0.00 ^Ac^
	5	0.28 ± 0.01 ^Cab^	0.30 ± 0.00 ^Da^	0.26 ± 0.01 ^Bb^	0.15 ± 0.01 ^Ab^
	7	0.27 ± 0.00 ^Ca^	0.31 ± 0.00 ^Da^	0.24 ± 0.01 ^Bab^	0.14 ± 0.01 ^Aa^
	9	0.29 ± 0.01 ^Cc^	0.31 ± 0.01 ^Da^	0.25 ± 0.00 ^Bab^	0.13 ± 0.00 ^Aa^

Note: The results are expressed as the mean ± SD. CG (without adding TPs), TP1 (0.01% TPs), TP2 (0.10% TPs), TP3 (0.30% TPs). Different shoulder-marked lowercase letters in the same column indicate significant differences between groups (*p* < 0.05). Capital letters with different shoulder labels indicate significant differences within groups (*p* < 0.05)

## Data Availability

The original contributions presented in the study are included in the article, further inquiries can be directed to the corresponding author.

## Supplementary Materials

The following supporting information can be downloaded at https://www.mdpi.com/article/10.3390/foods14132259/s1. Figure S1: Hydrophobicity of control and experimental groups under six freeze–thaw conditions; Table S1: Changes in the secondary structure of minced lamb after freeze–thaw cycles gel treated with tea polyphenols; Table S2: Number of I850/I830, Nburied and Nexposed in control and experimental groups under six freeze–thaw conditions; Table S3: Number of disulfide bonds in control and experimental groups under six freeze–thaw conditions; Table S4: Changes in thermal denaturation temperature of minced lamb after freeze–thaw cycles treated with tea polyphenols; Table S5: Changes in content of different water forms of minced lamb after freeze–thaw cycles before cooking treated with tea polyphenols; Table S6: Changes in content of different water forms of minced lamb after freeze–thaw cycles after cooking treated with tea polyphenols; Table S7: Changes in the emulsifying stability of minced lamb after freeze–thaw cycles treated with tea polyphenols [35,52,53].
