# Supplementary material for: The Effects of Tea Polyphenols on the Emulsifying and Gelling Properties of Minced Lamb After Repeated Freeze–Thaw Cycles
Source: Foods. 2025 Jun 26;14(13):2259. doi: 10.3390/foods14132259 (PMC12248921; doi:10.3390/foods14132259)
Supplement: Supplementary file 1 [file foods-14-02259-s001.zip › foods-3699115-supplementary.pdf]

Supplementary Materials

Effect of Tea Polyphenols on the Emulsifying and Gelling Properties of minced lamb after Repeated Freeze-Thaw Cycles

Table S1. Changes in the secondary structure of minced lamb after freeze-thaw cycles gel treated with tea polyphenols.

F-T cycles	Groups	α-helix/(%)	β-pleated sheet/(%)	β-turns/(%)	random coil/(%)
0	CG	53.90 ± 1.69^{Ac}	20.56 ± 3.11^{Aa}	8.37 ± 1.04^{Aa}	19.56 ± 2.11^{Aa}
	TP1	49.66 ± 4.25^{Ab}	23.42 ± 0.31^{Aa}	9.31 ± 0.75^{Ab}	22.60 ± 1.99^{Aa}
1	CG	50.40 ± 0.99^{Ab}	21.41 ± 2.17^{Aa}	8.00 ± 0.46^{Aa}	21.35 ± 1.60^{Aab}
	TP1	48.81 ± 1.70^{Ab}	24.02 ± 1.66^{Aa}	8.07 ± 0.74^{Aab}	23.87 ± 1.89^{Aa}
3	CG	46.69 ± 1.15^{Aa}	23.40 ± 0.95^{Aa}	6.68 ± 0.64^{Aa}	24.79 ± 2.45^{Ab}
	TP1	45.66 ± 3.33^{Aa}	25.69 ± 0.56^{Ba}	7.24 ± 0.88^{Aa}	26.50 ± 1.42^{Aa}
5	CG	49.89 ± 1.55^{Aab}	21.32 ± 2.06^{Aa}	7.13 ± 0.58^{Aa}	22.75 ± 1.86^{Aab}
	TP1	48.23 ± 3.03^{Ab}	22.96 ± 2.88^{Aa}	7.80 ± 0.71^{Aab}	24.27 ± 2.59^{Aa}
7	CG	49.71 ± 1.00^{Aab}	21.40 ± 1.02^{Aa}	7.32 ± 0.05^{Aa}	22.48 ± 0.47^{Aab}
	TP1	49.34 ± 3.17^{Ab}	22.94 ± 0.77^{Aa}	7.90 ± 0.30^{Aab}	23.94 ± 0.33^{Aa}
9	CG	49.95 ± 2.05^{Aab}	22.56 ± 1.47^{Aa}	7.85 ± 0.08^{Aa}	23.01 ± 0.53^{Aab}
	TP1	47.85 ± 0.35^{Ab}	23.28 ± 0.89^{Aa}	7.99 ± 0.60^{Aab}	24.29 ± 2.18^{Aa}

Table S2: Number of I850/I830, Nburied and Nexposed in control and experimental groups under six freeze-thaw conditions

F-T cycles	Groups	I850/I830	N_{buried}	$N_{exposed}$
0	CG	1.07 ± 0.02^{Aa}	0.24 ± 0.03^{Aa}	0.76 ± 0.03^{Aa}
	TP1	1.11 ± 0.04^{Aab}	0.18 ± 0.06^{Aab}	0.82 ± 0.06^{Ab}
1	CG	1.05 ± 0.01^{Aa}	0.27 ± 0.01^{Aa}	0.73 ± 0.01^{Aa}
	TP1	1.08 ± 0.04^{Aa}	0.22 ± 0.05^{Aabc}	0.78 ± 0.05^{Aab}
3	CG	1.03 ± 0.02^{Aa}	0.29 ± 0.03^{Aa}	0.71 ± 0.03^{Aa}
	TP1	1.06 ± 0.01^{Aa}	0.25 ± 0.02^{Abc}	0.75 ± 0.02^{Aab}
5	CG	1.07 ± 0.02^{Aa}	0.24 ± 0.02^{Aa}	0.76 ± 0.02^{Aa}
	TP1	1.16 ± 0.04^{Bb}	0.29 ± 0.01^{Ac}	0.71 ± 0.01^{Aa}
7	CG	1.05 ± 0.04^{Aa}	0.26 ± 0.05^{Aa}	0.74 ± 0.05^{Aa}
	TP1	1.07 ± 0.01^{Aa}	0.26 ± 0.04^{Ac}	0.74 ± 0.04^{Aa}
9	CG	1.05 ± 0.02^{Aa}	0.27 ± 0.02^{Ba}	0.73 ± 0.02^{Aa}
	TP1	1.13 ± 0.02^{Bab}	0.16 ± 0.03^{Aa}	0.84 ± 0.03^{Aab}

A value of I850/I830 between 0.5 and 1.25 indicates the presence of both an "exposed" and a "buried" state of hydrogen bonding. The formulas $N_{buried} + N_{exposed} =$

1 and $0.5N_{buried} + 1.25N_{exposed}d = I850/I830$ can be employed to ascertain whether the tyrosine residue is in the "buried" or "exposed" state.

Figure S1: Hydrophobicity of control and experimental groups under six freeze-thaw conditions

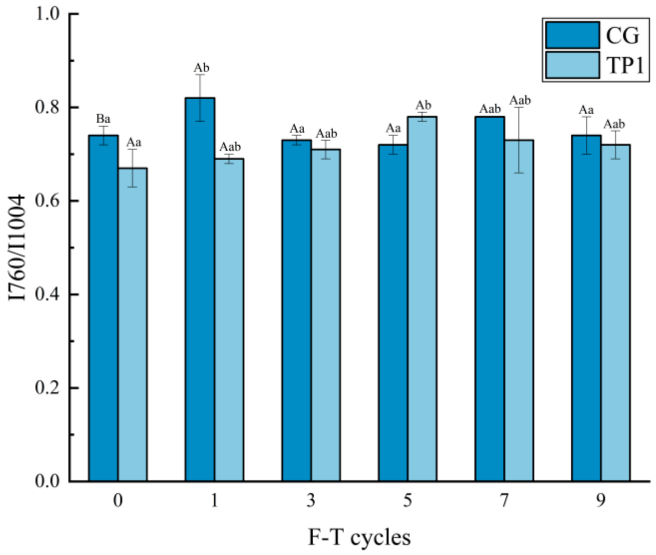

The vibrations observed at approximately 760 cm^{-1} can be attributed to the telescopic vibrations of tryptophan residues. These vibrations reflect alterations in the hydrophobic interactions of the protein, as indicated by the normalized intensities. The increase of hydrophobicity is primarily attributable to the alterations in the secondary structure of proteins resulting from freeze-thaw treatments, which facilitate the exposure of the hydrophobic aliphatic and aromatic amino acid side chain groups of denatured proteins on the surface of the molecules.And the decrease of hydrophobicity is primarily attributable to the free radical scavenging capacity of the 2-phenylbenzopyran structure of tea polyphenols in conjunction with hydroxyl groups. This capacity enables the inhibition of oxidative denaturation of proteins and the reduction of hydrophobic groups exposed on the surface of proteins (Mutilangi et al.,

1996) [1].

Table S3: Number of disulfide bonds in control and experimental groups under six freeze-thaw conditions

F-T cycles	Groups	I475/I1003	I514/I1003	I540/I1003
0	CG	0.79±0.05^{Aa}	0.79±0.06^{Aa}	0.81±0.04^{Aa}
	TP1	0.71±0.05^{Aa}	0.71±0.05^{Aa}	0.71±0.07^{Aab}
1	CG	0.92±0.08^{Bb}	0.89±0.07^{Bb}	0.90±0.08^{Bb}
	TP1	0.71±0.02^{Aa}	0.68±0.01^{Aa}	0.68±0.01^{Aa}
3	CG	0.79±0.02^{Ba}	0.79±0.01^{Ba}	0.78±0.03^{Ba}
	TP1	0.71±0.03^{Aa}	0.73±0.02^{Aa}	0.72±0.01^{Aab}
5	CG	0.81±0.03^{Aa}	0.80±0.04^{Aa}	0.80±0.03^{Aa}
	TP1	0.77±0.01^{Aa}	0.78±0.03^{Aa}	0.78±0.03^{Ab}
7	CG	0.85±0.01^{Aab}	0.82±0.00^{Aa}	0.82±0.02^{Aab}
	TP1	0.78±0.08^{Aa}	0.77±0.07^{Aa}	0.79±0.07^{Ab}
9	CG	0.76±0.05^{Aa}	0.78±0.05$_{Aa}$	0.76±0.05^{Aa}
	TP1	0.71±0.03^{Aa}	0.72±0.02^{Aa}	0.71±0.02^{Aab}

Table S4: Changes in thermal denaturation temperature of minced lamb after freeze-thaw cycles treated with tea polyphenols

Groups	F-T cycles					
	0/°C	1/°C	3/°C	5/°C	7/°C	9/°C
CG	130.02±1.31^{Ba}	130.51±4.66^{Aa}	144.07±6.57^{Ab}	131.67±1.45^{Aa}	133.32±1.68^{Aa}	134.33±2.29^{Aa}
TP1	124.82±0.30^{Aa}	128.80±2.49^{Aab}	139.58±2.73^{Ac}	130.63±1.80^{Ab}	130.88±1.31^{Ab}	130.65±1.74^{Ab}

Table S5: Changes in content of different water forms of minced lamb after freeze-thaw cycles before cooking treated with tea polyphenols

F-T cycles	Groups	P_{2b}/%	P_{21}/%	P_{22}/%
0	CG	2.29±0.49^{Aa}	2.89±0.19^{Abc}	94.43±0.93^{Aa}
	TP1	2.66±0.17^{Aa}	2.98±0.03^{Ab}	94.59±0.19^{Aa}
1	CG	2.48±0.16^{Ba}	3.11±0.04^{Ac}	94.38±0.18^{Aa}
	TP1	2.21±0.06^{Aa}	3.01±0.37^{Ab}	94.49±0.57^{Aa}
3	CG	2.21±0.18^{Aa}	2.84±0.08^{Abc}	94.74±0.39^{Aa}
	TP1	2.51±0.11^{Aa}	2.80±0.06^{Aab}	94.76±0.11^{Aa}
5	CG	2.36±0.13^{Aa}	2.93±0.11^{Abc}	94.70±0.41^{Aa}
	TP1	2.36±0.04^{Aa}	3.04±0.23^{Ab}	94.59±0.19^{Aa}

7	CG	2.59±0.26^{Aa}	2.45±0.23^{Aa}	95.02±0.22^{Aa}
	TP1	2.84±0.57^{Aa}	2.15±0.38^{Aa}	94.72±0.77^{Aa}
9	CG	2.52±0.27^{Aa}	2.75±0.14^{Aab}	94.54±0.49^{Aa}
	TP1	3.02±0.56^{Aa}	2.63±0.41^{Aab}	94.74±0.72^{Aa}

Table S6: Changes in content of different water forms of minced lamb after freeze-thaw cycles after cooking treated with tea polyphenols

F-T cycles	Groups	P_{2b} /%	P_{21} /%	P_{22} /%	P_{23} /%
0	CG	2.04±0.17^{Bb}	86.31±0.65^{Aab}	2.36±0.45^{Aa}	8.87±0.05^{Bbc}
	TP1	1.72±0.03^{Abc}	87.80±0.67^{Acd}	2.57±0.47^{Ad}	7.38±0.21^{Aa}
1	CG	1.73±0.30^{Aab}	87.91±2.19^{Abc}	2.22±0.72^{Aa}	8.06±1.25^{Aab}
	TP1	1.47±0.04^{Aa}	85.08±0.07^{Aa}	1.45±0.03^{Aa}	11.66±0.01^{Bc}
3	CG	1.77±0.08^{Aab}	89.43±1.30^{Ac}	1.87±0.00^{Aa}	7.69±0.00^{Ba}
	TP1	1.75±0.06^{Abc}	88.49±0.12^{Ad}	2.25±0.27^{Acd}	7.03±0.19^{Aa}
5	CG	1.79±0.20^{Aab}	88.40±0.09^{Bbc}	1.73±0.03^{Aa}	7.71±0.22^{Aa}
	TP1	1.71±0.03^{Ab}	87.12±0.35^{Abc}	1.90±0.26^{Aabc}	8.85±0.03^{Bb}
7	CG	1.71±0.11^{Aab}	86.78±0.56^{Ab}	1.77±0.17^{Aa}	9.34±0.25^{Ac}
	TP1	1.80±0.04^{Ac}	86.90±0.56^{Abc}	2.00±0.28^{Abc}	8.94±0.26^{Ab}
9	CG	1.67±0.10^{Aa}	84.37±0.24^{Aa}	1.72±0.07^{Aa}	12.24±0.07^{Ad}
	TP1	1.75±0.05^{Abc}	86.16±1.36^{Aab}	1.63±0.19^{Aab}	10.10±1.67^{Ab}

The changes in the peak area ratio of T2 relaxation time of minced meat after heating are demonstrated in the Table S7, and the integral area of the T2 interval of the minced meat gel as a percentage of the total integral area illustrates the relative content of moisture in each interval (Tornberg et al., 2000) [3].The relative content of P_{23} in CG was significantly lower than that in TP at 1 and 5 times of freezing and thawing ($P < 0.05$). The main reason was that the quinone formed by the oxidation of tea polyphenols reacted with the sulfhydryl groups in proteins to form “sulfhydryl-quinone” adducts, which impeded the formation of disulfide bonds between proteins, resulting in an increase in the relative content of free water lost from the minced lamb with tea polyphenols (Tazeddinova et al., 2022) [2].

Table S7: Changes in the emulsifying stability of minced lamb after freeze-thaw cycles treated with tea polyphenols

F-T cycles	CG/%	TP1/%	TP2/%	TP3/%
0	5.86±0.10Ac	7.76±0.32Bc	8.94±0.44Cc	11.62±0.08Dc
1	5.41±0.84Abc	8.57±0.62Bc	9.27±0.45Bc	11.15±0.53Cc
3	2.93±0.38Aa	4.40±0.16ABa	5.96±0.24BCa	7.08±0.87Ca
5	4.52±0.20Ab	5.73±0.06ABab	6.83±0.36Bab	9.07±0.99Cb
7	4.53±0.66Ab	5.89±0.17Bb	7.51±0.40Cb	9.64±0.14Db
9	4.47±0.44Ab	5.52±0.66Bab	7.17±0.35Cab	8.52±0.11Db

1. Mutilangi, W. A. M., Panyam, D., & Kilara, A. (1996). Functional Properties of Hydrolysates from Proteolysis of Heat-denatured Whey Protein Isolate. *Journal of Food Science*, *61*(2), 270-275. https://doi.org/https://doi.org/10.1111/j.1365-2621.1996.tb14174.x
2. Tazeddinova, D., Rahman, M. R., Bin Hamdan, S., Matin, M. M., Bin Bakri, M. K., & Rahman, M. M. (2022). Plant Based Polyphenol Associations with Protein: A Prospective Review [Review]. *Bioresources*, *17*(4), 7110-7134. https://doi.org/10.15376/biores.17.4.Tazeddinova2
3. Tornberg, E., Wahlgren, M., Brøndum, J., & Engelsen, S. B. (2000). Pre-rigor conditions in beef under varying temperature- and pH-falls studied with rigometer, NMR and NIR. *Food Chemistry*, *69*(4), 407-418. https://doi.org/https://doi.org/10.1016/S0308-8146(00)00053-4
